# Neural evidence supports a dual sensory-motor role for insect wings

**DOI:** 10.1098/rspb.2017.0969

**Published:** 2017-09-13

**Authors:** Brandon Pratt, Tanvi Deora, Thomas Mohren, Thomas Daniel

**Affiliations:** 1 Department of Biology, University of Washington, Seattle, WA 98105, USA; 2 Department of Mechanical Engineering, University of Washington, Seattle, WA 98105, USA

**Keywords:** flight control, campaniform sensilla, wings, strain sensing, Coriolis forces

## Abstract

Flying insects use feedback from various sensory modalities including vision and mechanosensation to navigate through their environment. The rapid speed of mechanosensory information acquisition and processing compensates for the slower processing times associated with vision, particularly under low light conditions. While halteres in dipteran species are well known to provide such information for flight control, less is understood about the mechanosensory roles of their evolutionary antecedent, wings. The features that wing mechanosensory neurons (campaniform sensilla) encode remains relatively unexplored. We hypothesized that the wing campaniform sensilla of the hawkmoth, *Manduca sexta,* rapidly and selectively extract mechanical stimulus features in a manner similar to halteres. We used electrophysiological and computational techniques to characterize the encoding properties of wing campaniform sensilla. To accomplish this, we developed a novel technique for localizing receptive fields using a focused IR laser that elicits changes in the neural activity of mechanoreceptors. We found that (i) most wing mechanosensors encoded mechanical stimulus features rapidly and precisely, (ii) they are selective for specific stimulus features, and (iii) there is diversity in the encoding properties of wing campaniform sensilla. We found that the encoding properties of wing campaniform sensilla are similar to those for haltere neurons. Therefore, it appears that the neural architecture that underlies the haltere sensory function is present in wings, which lends credence to the notion that wings themselves may serve a similar sensory function. Thus, wings may not only function as the primary actuator of the organism but also as sensors of the inertial dynamics of the animal.

## 1. Background

Animals rely on input from multiple sensory modalities to accomplish complex movement behaviours. From navigating in complicated habitats [1] to locating mates and avoiding prey [2,3], animals use visual, chemosensory, thermosensory, and mechanosensory information to coordinate motor commands for the task at hand [4–8].

Animal flight control, in particular, strongly depends on multisensory integration due in part to the inherent pitch instability associated with this mode of locomotion [9]. Unlike movement on land where multiple pairs of legs can provide stable support [10] or in water where instabilities have a vastly lower impact on movement control [11], flapping flight presents a stabilization challenge [9]. Whereas flight primarily relies on visual information, without which animals rarely fly, visual processing speeds are too slow to support rapid flight behaviours [12,13]. However, mechanosensory systems provide a rapid and parallel sensory processing pathway that compensates for slower visual systems [6,14–17].

In dipteran insects, halteres are thought to function as gyroscopic sensors that have the exquisite capacity to detect the Coriolis forces associated with body rotations [18–20]. By contrast, for the hawkmoth (*Manduca sexta*), a non-dipteran species that lacks halteres, Sane *et al.* [21] suggest that antennae also serve a similar mechanosensory function [21]. In both cases, removal of halteres or antennae compromises flight performance [21,22]. Moreover, electrophysiological data from primary afferents associated with haltere mechanosensors (campaniform sensilla) show rapid and precise encoding of the forces acting on them [14–16]. There is similar evidence for the mechanosensory afferents of the moth antennae [23].

**Figure 1.**
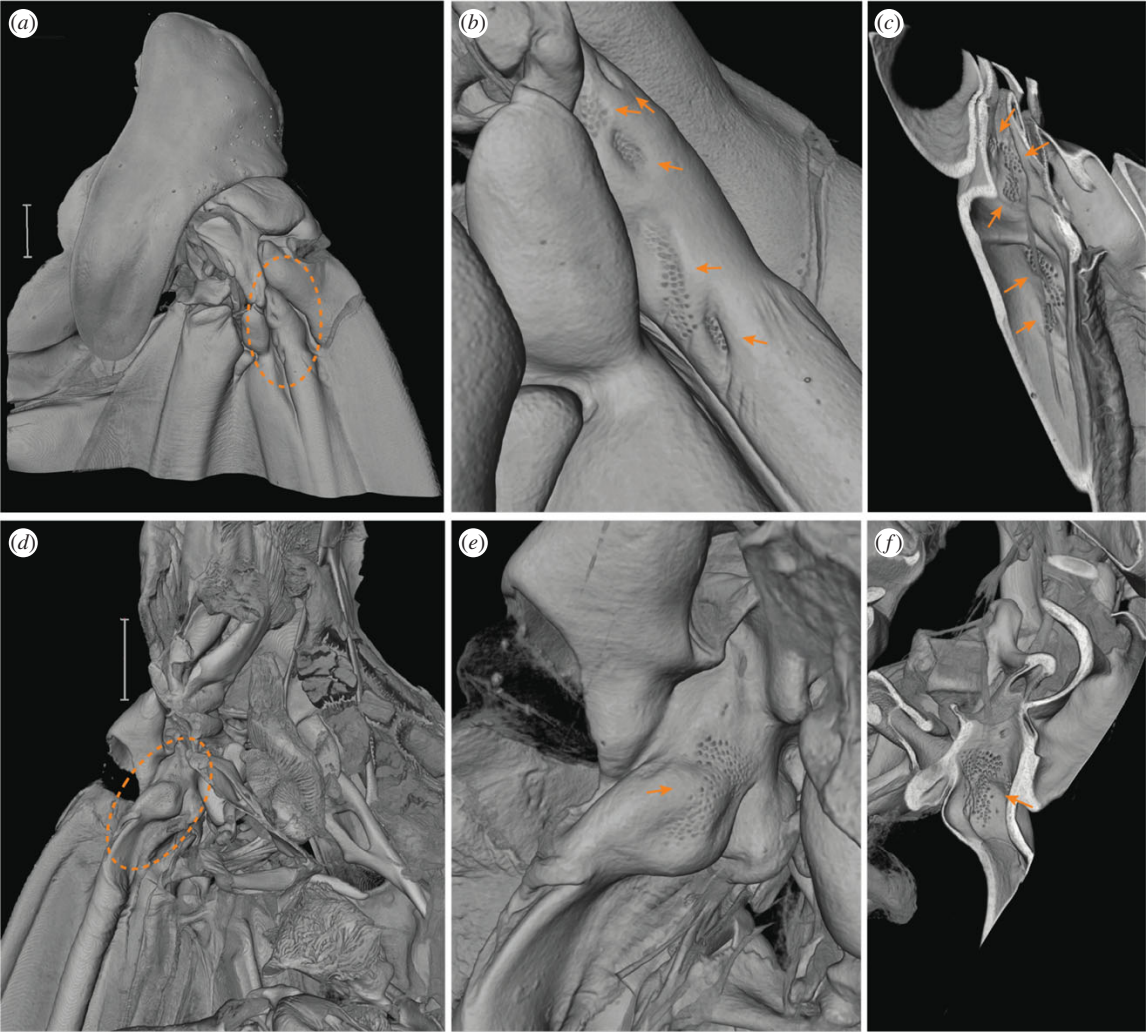
The wing surface contains multiple fields of campaniform sensilla. (*a*) Dorsal surface of the wing base with one sensilla-rich region highlighted (in dashed orange circle) and zoomed in (*b*,*c*). (*b*) Here, the campaniform sensilla occur in five distinct fields (orange arrows). (*c*) The same fields are visible from underneath, and can be viewed by virtually slicing the three-dimensional volume (orange arrows). (*d*) Ventral surface of the wing base with another sensilla field highlighted (in dashed orange circle) and zoomed in (*e,f*). (*e*) The highlighted sensilla field zoomed in and viewed on the ventral wing surface and (*f*) the ventral sensilla field is also visible by virtually slicing the three-dimensional volume to reveal it on a dorso-lateral view. Scale bar in (*a*) and (*d*) = 1 mm, voxel size 2.94 μm. (Online version in colour.)

Halteres are evolutionarily derived from insect wings. For selection to act on wings in ways that could give rise to halteres, one would expect wings themselves to provide some level of information about Coriolis forces. Like halteres, wings contain numerous campaniform sensilla, some distributed over the surface of the wing and others arranged in patches near the base (figure 1, electronic supplementary material, movie S1) [24,25]. Biomechanical analyses showed that torsional deformations occur as a result of Coriolis forces acting on flapping wings during body rotations [26]. Moreover, recent behavioural evidence showed that in addition to visual information, hawkmoths use wing mechanosensory information to elicit postural changes associated with flight control [27].

With mounting evidence that wings themselves serve a sensory function similar to halteres, we asked if wings have the neural architecture in place to facilitate a gyroscopic function [28–30]. Here, we seek to characterize the encoding properties of wing campaniform sensilla to explore their similarity to haltere sensilla. Based on what is known about halteres [16], we hypothesize that (i) wing campaniform sensilla, like haltere neurons*,* encode mechanosensory information rapidly and precisely and (ii) there is a diversity in the encoding properties of the wing campaniform sensilla. Thus, in addition to the pure mechanosensory role ascribed to halteres, wings may serve the dual roles as both sensors and actuators.

## 2. Material and methods

### (a) Animal preparation

**Figure 2.**
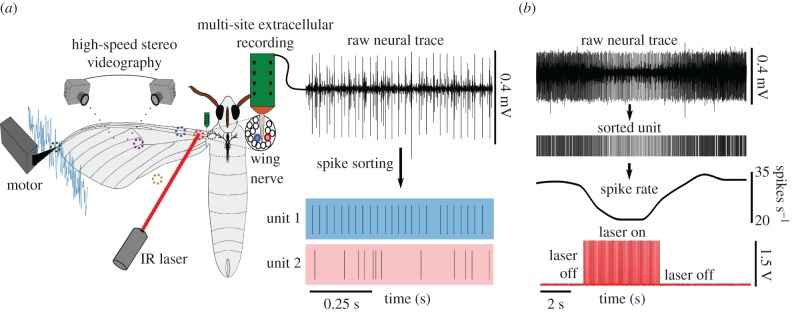
Experimental set-up for mechanically stimulating the wing campaniform sensilla in tandem with focal IR heating to identify their receptive fields on the wing surface and extract their encoding properties. (*a*) An anaesthetized moth was held in a custom-built immobilization chamber (not shown here) abducting the wing with the ventral surface facing up. A motor lever arm provided mechanical stimulus to the moth wing tip via a plastic grasp while simultaneously recording the wing movements using two high-speed video cameras at 1 000 frames per second (fps) allowing us to reconstruct wing displacement at specific locations on the wing. We used a multi-site extracellular electrode to record the resulting neural activity from the right forewing nerve. The extracellular neural data was spike sorted to reveal the activity of individual neuronal units (two sorted units are shown). Before applying the mechanical stimulus, we used focal IR laser heating at specific locations on the wing surface (dotted circles) while recording from the forewing nerve to reveal each neuronal unit's receptive field location on the wing. (*b*) A representative wing base-localized unit showing a reduction in firing rate (upper trace in black) during focal heating (lower trace). (Online version in colour.)

All recordings were performed at 25°C (room temperature) on 1–3 days post-eclosion adult hawkmoths, *M. sexta*, (*N* = 33, 16 males and 17 females) obtained from a colony maintained at the University of Washington. Moths were collected in individual containers with a moist tissue to prevent desiccation. The moths were then anaesthetized at 4°C for approximately 24 h. They were prepared for recordings by removing the legs, the left-wing pair, and the right hindwing. We descaled the cuticle around the base of the right forewing and painted white dots on the ventral wing surface using white acrylic paint. The dots were painted along the leading edge, the wing base, and between the wing veins to be used as markers for reconstructing the time-varying changes in wing displacement arising from mechanical stimuli. Moths were anaesthetized at 4°C for another approximately 24 h before recording from the wing nerve. They were then placed in a custom designed three-dimensional printed immobilization holder such that the right forewing was abducted at approximately 90° from its normal resting position, with the ventral surface facing up for neural recordings (figure 2*a*). We exposed the right forewing nerve by removing the right tegula, basalare, and the trachea and other soft tissue overlying it. Two to three drops of physiological saline solution (150 mM NaCl, 3 mM CaCl_2_, 3 mM KCl, 10 mM *N*-Tris [hydromethyl methyl]-2-aminoethanesulfonic acid buffer, and 25 mM sucrose, pH 6.5–7.5) [31] were applied to the nerve to prevent desiccation.

### (b) Experimental procedure

#### (i) Electrophysiological recordings with simultaneous high-speed videography

We penetrated the right forewing nerve with a 16-channel extracellular electrode (model: A1 × 16-3 mm-25-177-A16, NeuroNexus, Ann Arbor, MI, USA). Neural signals were filtered and amplified (300–1 000 Hz bandpass, 1 000-fold amplification) using an extracellular amplifier (model 3600, A-M systems, Sequim, WA, USA) and recorded at 40 kHz using a data acquisition board (Model NI USB-6259, National Instruments, Austin, TX, USA). A tungsten wire, inserted through the cuticle on the lateral thorax, served as the reference electrode. In a typical preparation, we were able to maintain stable recordings of spiking activity in up to three simultaneous channels for more than 2 h.

We recorded the neuronal activity as we delivered mechanical stimuli to the wing tip through a motor lever arm (Model 305B, serial number 305034, Aurora Scientific Inc., Aurora, ON, Canada) via a plastic clasp. Custom Matlab code (Mathworks Inc., Natick, MA, USA) controlled the motor lever system to deliver three bouts of 25 Hz sinusoidal stimuli for 4 s, each of progressively larger wing tip amplitudes (peak to peak of 4.4, 8.8, and 13.2 mm, measured to the nearest 0.1 mm via an ocular micrometer). This was followed by 30 10 s bouts of band-limited (2–300 Hz) Gaussian white noise (hereafter white noise) of a maximum amplitude of 9.5 mm. There was a rest period of 1 s between the sinusoidal and the white noise segments, and a 2 s rest period between every 10 repeats of white noise stimuli. We sorted spikes to identify neuronal units using all channels that showed neural activity with NeuroExplorer^®^ (V. 5, Nex Technologies, Madison, AL, USA) and Offline Sorter (V. 4, Plexon Inc., Dallas, TX, USA), using threshold and time alignment based on the spike peak, and sorted using PCA. The three axes of the PCA were based on two dominant principle components and the spike width. Spike time data were imported into Matlab for further analyses. During the first 10 s presentation of the white noise mechanical stimulus, we recorded the three-dimensional position of wing markings using two high-speed video cameras (Miro-4M VR0308 and VR711, Vision research, Wayne, NJ, USA at 320 × 240 resolution, 1 000 fps and 200–400 ms exposure) to compute wing region-specific mechanosensory stimuli (local displacements).

The high-speed videos of two male and two female wings were digitized and used to reconstruct the vertical displacement at their wing base (for an example of high-speed video and three-dimensional reconstruction see electronic supplementary material, movie S2). The reconstruction was done using Hedrick's custom software in Matlab [32]. We used digitized data from these four moths to compute a transfer function (in gain and phase) relating displacements of the wing tip to wing base. We used this transfer function for units that were localized to the base (see below).

#### (ii) Laser-based focal heating identifies wing regions corresponding to campaniform receptive fields

Because all of the campaniform sensilla are mechanically coupled through the entire wing blade, using simple direct punctate mechanical stimuli makes localizing the receptive fields quite challenging during electrophysiological recordings. To address this issue, we developed a new method to alter the activity of campaniform sensilla without the complication of stress localization. We relied on a combination of thermal sensitivity of campaniform sensilla [33–35] and a focused IR laser to localize regions of the wing. While simultaneously recording from the wing nerve using methods detailed above, we focally heated specific locations on the wing surface with an IR laser (785 nm, 8 mW, model CDL-3144-008S laser diode, beam diameter of 0.5 mm, Sanyo, Japan). A custom-built Arduino circuit controlled the laser (figure 2*b*). Each location was heated five times at a duty factor of 25% for 5 s (100 pulses of 50 ms each). The duty factor was selected to provide a robust thermal response without damaging the wing. The input pulse to the laser was recoded along with the corresponding neural data on the data acquisition board at 40 kHz. Changes in the unit's firing rate during focal heating were used to localize the receptive field of that unit on the wing (see electronic supplementary material, figure S1 for details of how we classified thermally sensitive units).

### (c) Computational analyses of the neuronal data

#### (i) Feature detection (spike-triggered averages and nonlinear decision functions)

We analysed only those identified units which had high stimulus-response coherence (high mutual information [36], for details of this process see the electronic supplementary material). Using methods similar to previous studies [16,37], we computed the spike-triggered ensemble (STE) for each unit by selecting the stimulus history in the 40 ms time window immediately preceding each spike. Taking the mean of the STE yielded the spike-triggered average (STA). We also calculated the prior stimulus ensemble (PSE) by randomly selecting timestamps throughout the white noise stimulus period, and similarly selecting the 40 ms stimulus history preceding each of those timestamps. The total number of timestamps for generating the PSE was the same as the recorded number of spikes for each unit.

To characterize the nonlinear decision function (NLD), we computed a histogram of the projections of the STA onto the stimulus for each spike (each element of the STE) with a bin width of 0.4 of the standard deviation of the PSE. We normalized this histogram by dividing each bin by the product of the norm of the STA and each element of the STE. Similarly, we constructed and normalized a histogram of the projections of the STA onto each element of the PSE. We then constructed the NLD histogram by dividing the normalized histogram built from the STE by the normalized histogram built from the PSE. This histogram was converted into predicted spike rate by multiplying it by the mean firing rate of the unit during the white noise stimulus period. We then performed a cubic spline interpolation between the predicted spike rates of each bin to fit a curve onto this histogram.

Normalizing in this manner ensured that the NLD had a value between −1 and 1. The NLD represents the selectivity of a unit in response to any particular stimulus.

#### (ii) One-dimensional spike rate prediction model

Using the STA and NLD of a unit, we predicted its spike rate in response to the three different amplitudes (4.4, 8.8, and 13.2 mm) of sinusoidal stimuli. We convolved the STA with the 40 ms cycle of the sinusoidal stimuli and normalized the projection value in a similar manner to the NLD construction. For each projection value, we used the NLD to calculate the corresponding predicted spike rate. We compared the predicted spike rate with the recorded spike rate by reconstructing a recorded spike rate histograms. This recorded spike rate histogram was built by binning over a 1.25 ms time window and averaging the spike rate across the 100 repeats of a single 40 ms sinusoidal cycle. To compensate for binning artefact, we further convolved the histogram with a 40 ms Gaussian window (*σ* = 5.3 ms). We calculated the root-mean-square error between the predicted spike rate and the fitted recorded spike rate as a measure of how well the one-dimensional spike rate prediction model predicts a unit's spike rate during the sinusoidal stimuli.

## 3. Results

We acquired multi-channel extracellular recordings of the wing afferents from a total of 33 hawkmoths, while delivering mechanical stimuli to the wing tip. We identified 95 wing mechanosensory units using offline spike sorting techniques (figure 2*a*; *Material and Methods*). To study the encoding properties of these campaniform sensilla, it was essential to approximate the local mechanical stimulus experienced by each sensillum during perturbations to the wing tip. Hence we drew upon the thermosensitive properties of mechanosensors and developed a novel laser-based focal heating method to localize the receptive field of each afferent. We captured high-speed videos (1 000 fps) of the wing during mechanical stimulation to reconstruct the local displacements at different regions on the wing.

### (a) Focal heating reveals local displacements for identified receptive fields

Focal heating combined with multi-channel extracellular recording and high-speed videography allowed us to reconstruct the local displacements for identified receptive fields. In our experiments, most units showed a reduction in firing rate when heated (figure 2*b*) although a small subset of units showed an increase. This focal heating technique allowed us to localize 31 units to the wing base (for details see electronic supplementary material, figure S1 and methods). The remaining 64 units could not be localized to any region on the wing. Most of these units were not tonically active and hence we could not observe changes in firing rate during local heating. We cannot rule out the possibility that the non-localized units also project from the wing base. We used the reconstructed wing displacement associated with the 31 units localized to the wing base (referred to as the *base-localized units)*, and the wing tip deflection for the other 64 units (referred to as the *non-localized unit*s) for further analyses.

From our calculations for the signal response (SR) coherence between the spike train of each unit and the motion stimulus (see electronic supplementary material for details on SR coherence analysis, figure S2), 14 out of the 31 base-localized mechanosensory units and 34 of the 64 non-localized mechanosensory units had a significant SR coherence at one or more frequencies (SR coherence of the unit's spike train and mechanical stimulus greater than 95% CI of a distribution of SR coherence of randomly permuted spike train and mechanical stimulus). These 14 base-localized and 34 non-localized units were used for further analyses using either the base or wing tip deflection as estimates of the mechanical stimulus driving their activity.

### (b) Wing mechanosensory units show rapid and selective encoding for a diversity of features

**Figure 3.**
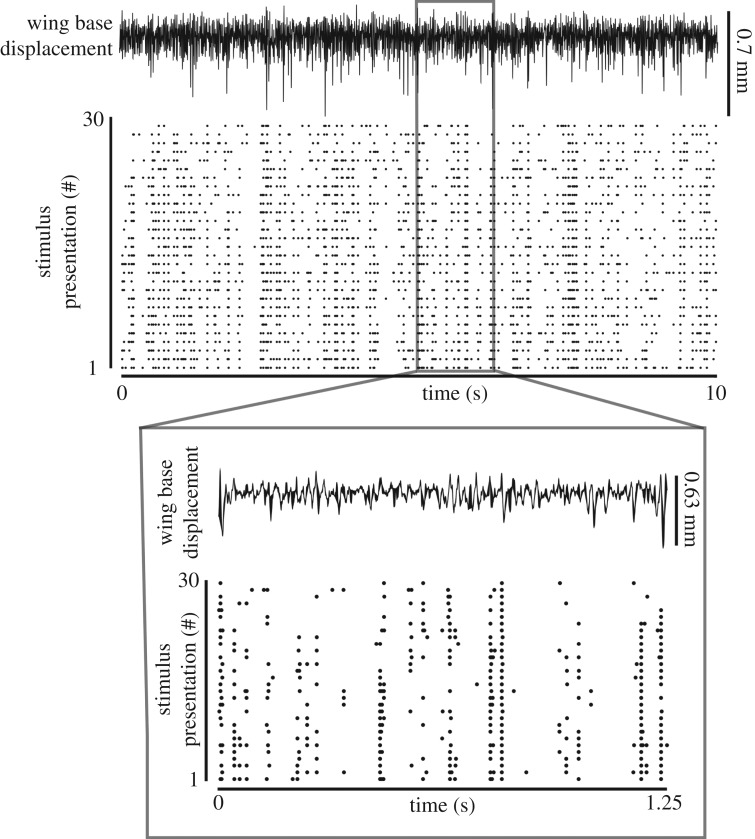
Forewing mechanosensory units fire reliably to repeated white noise mechanical stimuli. Raster plot showing the spike timing for a representative mechanosensory unit projecting from the wing base (*lower plot*; each dot represents a single spiking event) in response to repeated 10-s-long, band-limited (2–300 Hz) white noise mechanical stimuli delivered to the wing tip. Vertical displacement at the wing base estimated from high-speed stereo videography (*upper trace*).

**Figure 4.**
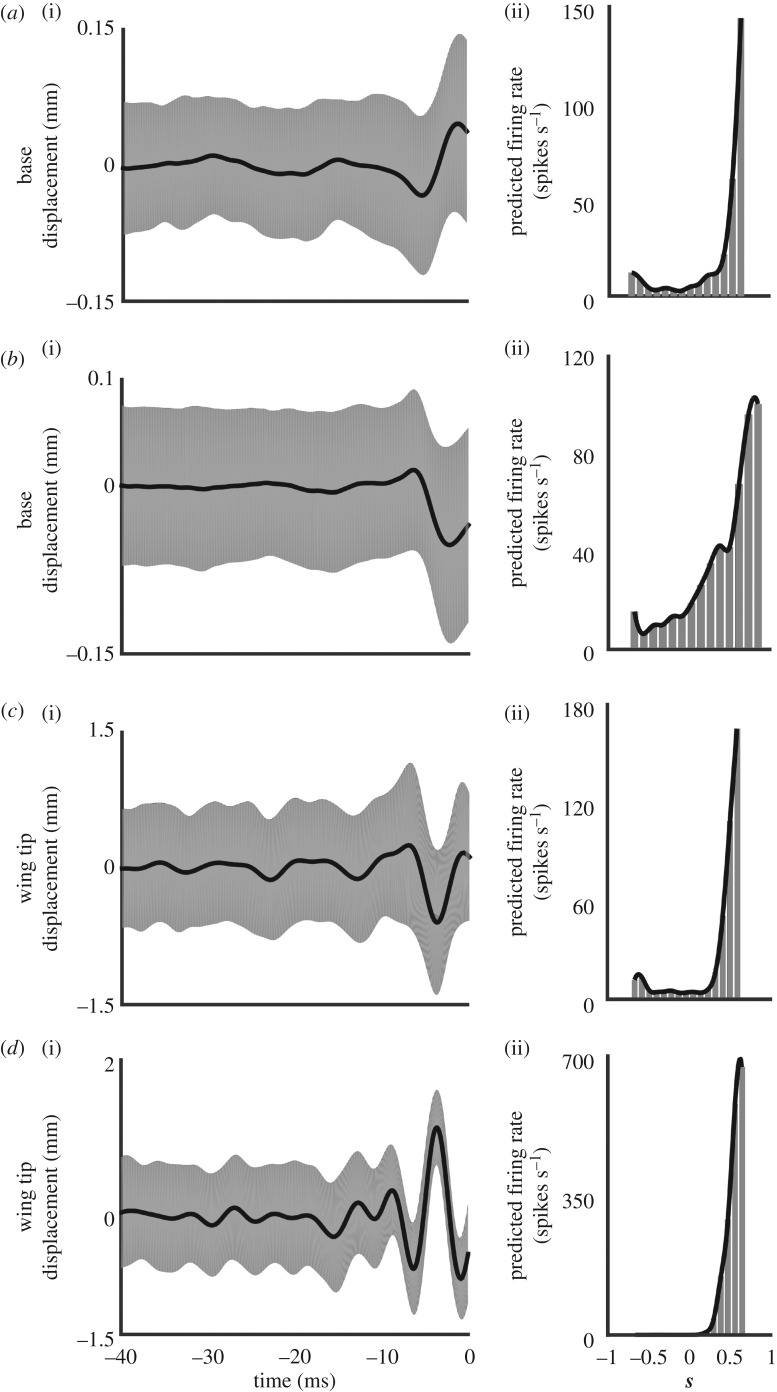
Wing mechanosensory units respond rapidly and selectively to diverse stimulus features as shown by their STAs and nonlinear decision functions (NLDs). STAs (*a*(i), *b*(i)*, c*(i), *d*(i) STA in black ± s.d. in light grey) for two representative wing mechanosensory units projecting from the wing base (*a*,*b)*) and two representative units not localized to a region on the wing (*c*,*d*) are plotted for the 40 ms prior to spike occurrences. These units show that stimulus motions within 10 ms yield spikes. The NLDs (*a*(ii), *b*(ii)*, c*(ii), *d*(ii) grey histogram of the predicted spike rate over a range of stimulus projections and the fitted curve in black) for each of the representative mechanosensory units is plotted as the predicted firing as a function of the similarity of the stimulus to the STA. That similarity (***s***) is computed by the projection STA onto the stimulus and was normalized to their respective amplitudes. The sharp rise in the NLD as the ***s*** tends to 1 shows that the unit is highly selective for stimulus features that resemble the STA.

To identify how wing campaniform sensilla encode mechanical stimuli, we analysed the response to 10 repeats of 10-s-long, band-limited (2–300 Hz) white noise mechanical stimulus. The consistency of the unit's spike timing during the 30 white noise repeats (figure 3) suggests strong stimulus feature selectivity. We used the STA to compute the feature of wing displacement driving neural activation. Different units have varying STA shapes indicating diversity in the encoding properties of wing campaniform sensilla (figure 4*a*(i)(ii)*, b*(i)(ii); base-localized units, figure 4*c*(i)(ii), *d*(i)(ii); non-localized units). The amplitude of the base-localized unit's STA reveals that these units respond to stimuli at least as small as 0.15 mm (figure 4*a*(i), *b*(i)). To determine how rapidly these units encode mechanical information, we measured the mean time at which the STA reached its maximum absolute value displacement from rest. The latency of firing (shown as means ± s.d.) for the base-localized units was 1.9 ± 2.1 ms (*n* = 14) and 2.8 ± 1.8 ms (*n* = 34) for the non-localized units. Another measure for latency is the time at which the standard deviation of the STA is at its minimum. This latter metric indicates the time prior to a stimulus when the motion amplitude and its time history most reliably lead to a response. Both are fairly similar measures of the timing relative to a spike. Using this measure, the latency for the base-localized units was 10.6 ± 10.5 ms (*n* = 14) and 5.1 ± 1.6 ms for the non-localized units (*n* = 34). Thus, the mechanosensory units showed low latency spike timing to specific features of the wing displacement.

We examined all of the STAs using singular value decomposition (SVD), which, like a PCA, extracts the dominant characteristics of the stimulus waveforms that initiate spikes (see electronic supplementary material for details and methods on the SVD analysis, figures S3 and S4). Two dominant modes weighted for their singular values show similar shapes with similar latencies and contain most of the energy of stimulus features that drive responses. The second mode resembles the derivative of the first dominant mode. In general, all STAs show similar latencies to that of the dominant modes.

We constructed the one-dimensional NLDs of these units as a measure of their selectivity for particular stimulus features (figure 4*a*(ii), *b*(ii), *c*(ii)*, d*(ii)). The shape of the NLD is a measure of the unit's selectivity. We characterized the selectivity of identified units by calculating the value of the normalized stimulus projection at half of the maximum predicted spike rate: more selective units have a higher value for the projected half maximum. The normalized projection value at half maximum was 0.18 ± 0.45 for the base-localized units (mean ± s.d., *n* = 14) and 0.44 ± 0.12 for the non-localized units (mean ± s.d., *n* = 34). Comparing these to the stimulus projection value at the largest predicted firing rate (0.59 ± 0.36 for the 14 base-localized units and 0.59 ± 0.09 for the 34 non-localized units) shows strong selectivity.

### (c) The one-dimensional spike rate model constructed from white noise stimuli predicts a unit's spike rate to novel sinusoidal stimuli

**Figure 5.**
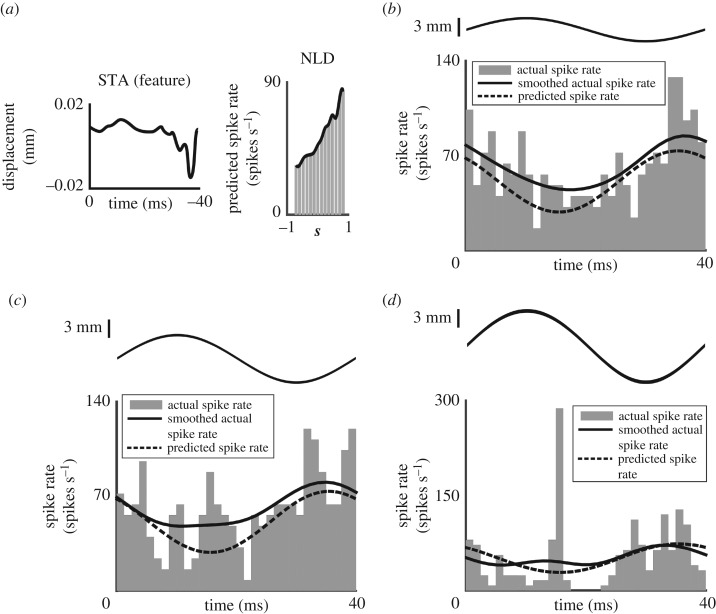
The one-dimensional spike rate prediction model extracted from the white noise mechanical stimulus captures the response of wing mechanosensory units to a novel sinusoidal stimuli. (*a*) The STA and NLD for a representative wing base unit were used to predict its spike rate in response to a 25 Hz sinusoidal wing displacement of varying amplitudes (*b*) 4.4 mm; (*c*) 8.8 mm and (*d*) 13.2 mm, black lines above each panel. The predicted spike rate (the black dashed line) closely resembles the recorded spike rate (black line). The recorded spike rate histogram is represented by grey bars. The predicted spike rates match measured spike rates more reliably at lower amplitudes. We used 100 repeats of sinusoidal mechanical stimuli at each amplitude to reconstruct the average recorded spike rate.

We predicted the response of a unit to 25 Hz sinusoidal stimuli using the STA and NLD constructed from the white noise mechanical stimuli (figure 5 and for raw neural activity during the sinusoidal displacement, see electronic supplementary material, figure S5). The predicted spike rate (black dashed line) captures the shape of the smoothed recorded spike rate (solid black line) for a representative unit over one sinusoidal cycle (average spike rate histogram binned over 1.25 ms across 100 repeats; in grey). This spike rate prediction model faithfully predicts the unit's recorded spike rate during sinusoidal displacements as shown by a relatively small root-mean-square error between the predicted and recorded spike rates (see electronic supplementary material, figure S6 and table S1). The smaller amplitude sine wave corresponds to the energy present at that frequency in the white noise stimulus used to extract the STA. That lower amplitude sine stimulus shows closer agreement between measurement and prediction than is the case for higher amplitude stimuli. All three amplitudes show a peak of spike activity shortly following the minimum displacement. At higher amplitudes (figure 5*c*,*d*) spike activity appears at a time shortly after the maximum displacement of the sine stimulus.

## 4. Discussion and conclusion

Four key findings emerged from this study. First, the primary afferents projecting from campaniform sensilla, both those localized at the wing base and those that could not be localized, showed rapid responses to mechanical stimuli, often within approximately 2 or 3 ms. Second, there is variation in the shape of the stimulus features (STA) that drive responses. Third, the shapes of the NLD indicate strong selectivity for particular stimulus features. Fourth, using the STAs and NLDs of the wing mechanosensory units, we were able to predict the empirical spike rates during sinusoidal displacements of varying amplitudes to the wing. These results provide strong neural evidence for the similarity in the encoding properties between wing and haltere mechanosensory neurons. Indeed, nearly all of these results recapitulate those found by Fox *et al.* [16] for haltere neurons, which also encode mechanosensory information rapidly and precisely. In that study, 36 recorded haltere neurons responded in 3.0 ± 2.8 ms to specific stimulus features [15,16], and therefore, had rapid and precise spike timing. This corresponds with our results from wing mechanosensory neurons. Haltere neurons also demonstrated variability in their encoding properties with regards to the shape of their STAs [16] in ways that strongly resemble the variation we note for wing campaniform sensilla. Haltere neurons were further found to be highly selective for particular stimulus features as indicated by their NLDs [16]. Collectively, these similarities suggest that the neural equipment encoding mechanosensory information is common to both wings and halteres. Therefore, like halteres, wings may serve a similar sensory function [18]. Most importantly, these data support the ideas raised by Eberle *et al.* [26] and Dickerson *et al*. [27] that wings may serve a role as sensors of body dynamics.

While we have strong evidence for this dual actuator and sensor role for wings, our estimates of the strain experienced by any one campaniform sensillum require technology with vastly higher spatial and temporal precision than afforded by our current methods involving multi-camera high-speed videography and three-dimensional reconstruction. For the 34 units that we could not spatially localize on the wing, we used the vertical displacement of the wing tip as a proxy for the strains that these sensilla experienced during mechanical perturbations to the wing. Prior studies of haltere neurons were also limited in their inability to reconstruct the precise stimuli experienced by a single sensillum [16]. Insect wings are composed of a complex matrix of rigid veins that provide stiffness to the wing and thin cuticular sheets that are flexible and folded into various complex shapes. Any motion delivered to the wing tip is filtered by the wing's shape and spatial stiffness distribution such that local regions on the wing surface might experience different strains; altered both in phase and amplitude (see electronic supplementary material, movie S2) [26,38,39]. Knowing the strain at the level of a single campaniform sensillum will reveal a more proximate estimate of the feature detection for these sensors, and a potentially better understanding of the variation found among their encoding properties [40]. An important caveat is that precise calculation of strain requires equally precise information about details of the cuticular structures surrounding and including each sensillum. Thus we believe larger scale estimates of mechanical stimuli are sufficient. Moreover, our ability to use measured STAs and NLDs to reconstruct observed neural responses lends credence to this approach.

Drawing on the thermosensitive properties of campaniform sensilla permitted a novel method for localizing receptive fields in an extracellular recording preparation. However, a second limitation in this work is that only tonically firing units could be localized. Thus, we examined a subset of the possible units that may contribute to wing sensing. Nevertheless, the subset of units shows sufficient similarity to the range and quality of responses found in haltere neurons to justify this approach.

Because we used extracellular recording techniques, we can never be 100% certain that spikes that are sorted and clustered correspond to a particular neuron (campaniform sensillum). For intracellular recording, we would be far more confident, as was the case for Fox *et al*. [16], that the recorded spikes were from a single neuron. As it is, we used two leading principal components (including timing coincidence on multiple channels, and spike width) and the peak-to-valley amplitude. While this is a stricter classification process than one using equivalent spike amplitudes, there is a remote possibility that more than one sensillum responded at the same time with the same spike shape. Additionally, we used a fairly stringent test to select only those units whose signal to response coherences was, via bootstrap methods, statistically significant. This metric, together with our spike sorting algorithms, makes us fairly confident that we are dealing with single campaniforms.

As mentioned above, the STA and NLD derived from a white noise stimulus was used to predict spike responses to the more physiologically relevant mechanical stimulus of a 25 Hz sine stimulus (figure 5). That prediction, however, was less effective at stimulus amplitudes that were higher than those associated with the white noise stimulus. Our ability to deliver larger amplitude white noise stimuli was limited by the total energy we could impart to the wing without incurring significant damage, likely a consequence of the energy at the higher frequencies. That said, the relationship between predicted and measured spike rate was fairly robust for the spiking associated with the peak downward (minimum) wing deflection stimulus. At the peak upward stimulus, the unit activity, initially relatively modest at low amplitudes, begins to appear at larger amplitudes. This may be a consequence of a feature of insect mechanoreceptors that demonstrate responses to both stimulus onset and offset (e.g. [40]).

Despite these limitations, prior work [26,27] and our current results provide strong evidence that wings could serve a function in sensing inertial dynamics of the body. Indeed, because halteres are derived from wings, evolution suggests that the function of gyroscopic sensing in halteres was likely present in such ancestral structures. Eberle *et al*. [26] previously demonstrated that the torsion arising when a flapping and flexing wing experiences rotational forces could lead to changes in the pattern of strain over the surface of the wing. They further suggested that torsion would influence the spatial and temporal pattern of neural activity of wing campaniform sensilla. Additionally, behavioural results from Dickerson *et al*. [27] showed that mechanical stimulation to wings drove stabilization reflexes. Together, the results from Eberle *et al*. [26] and Dickerson *et al*. [27] support the idea that wings function as sensors of body dynamics. Here, we add additional support to this idea by highlighting the similarities in the encoding properties of wing and haltere campaniform sensilla [16].

There is still more to understand at the circuit level about how mechanosensory information is processed, integrated, and transformed into behavioural outputs [40,41]. It may be that similar research on a range of taxa can reveal common encoding properties and possible functions of wing campaniform sensilla, especially given the immense diversity in wing morphology across all insect taxa [38]. Additionally, how campaniform sensilla are distributed over the wing blade and how that distribution varies taxonomically remains an open issue [24,42]. Indeed, recent studies of optimal sensor placement in a few taxa show that flapping dynamics are best detected with concentrations of campaniforms at the base [43]. However, more complex dynamics, such as body rotations or accelerations in various axes will drive even more complex wing deformations. Those deformations, filtered through the neural responses of a distribution of sensors, could be used to classify or measure body rotations and accelerations about multiple axes.

## Supplementary Material

Supplemental Metods and Analysis

## Data, code, and materials

All data are uploaded into the Dryad system: http://dx.doi.org/10.5061/dryad.7hm1m [44]. Code is available on Github at https://github.com/Prattbuw/CODE-Neural-evidence-supports-a-dual-sensory-motor-role-for-insect-wings-Pratt-et-al.#code-neural-evidence-supports-a-dual-sensory-motor-role-for-insect-wings-pratt-et-al.
