# Supplementary material for: Neural evidence supports a dual sensory-motor role for insect wings
Source: Proc Biol Sci. 2017 Sep 13;284(1862):20170969. doi: 10.1098/rspb.2017.0969 (PMC5597827; doi:10.1098/rspb.2017.0969)
Supplement: Supplemental Metods and Analysis [file rspb20170969supp1.pdf]

Supplementary Material:

Neural evidence supports a dual sensory-motor role for insect wings

Brandon Pratt[1], Tanvi Deora[1], Thomas Mohren[2], and Thomas Daniel[1,2]

Department of Biology[1],

Department of Mechanical Engineering[2],

University of Washington

Seattle 98105 WA

USA

Supplementary Figures:

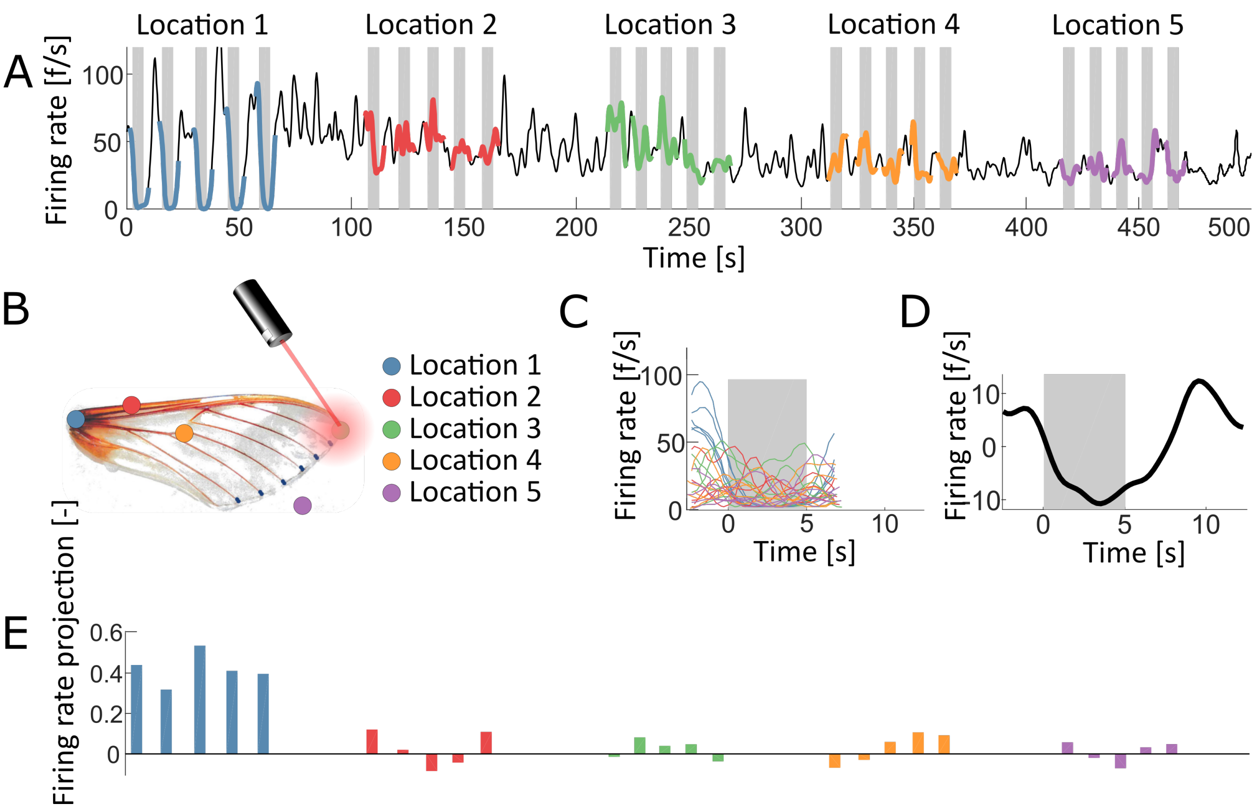

Supplementary Figure 1: Focal heating revealed the receptive field of individual neuronal units. (A) Firing rate of a unit was calculated during focally heating (gray bars) of different regions of the wing. The colored portion of each firing rate record corresponds to the time window in which we sampled the firing to determine the change in firing rate due to heating. The color corresponds to location on the wing shown in panel B. That window width is also shown in panels C and D where the firing rate for all . Each location was heated five times and the resulting 25 firing rate responses across all locations (color corresponds to location) during focal heating were collected (C), and averaged (D). (E) We calculated the projection of the individual firing rates onto the average. If the resulting projection values were large and coherent across the five repeats of focal heating for a particular wing location, then the unit was classified as projecting from that location. For instance, the representative unit shown here coherently responds when the wing base is focally heated as compared to the other heating locations, and therefore projects from the wing base.

Supplementary Figure 2:

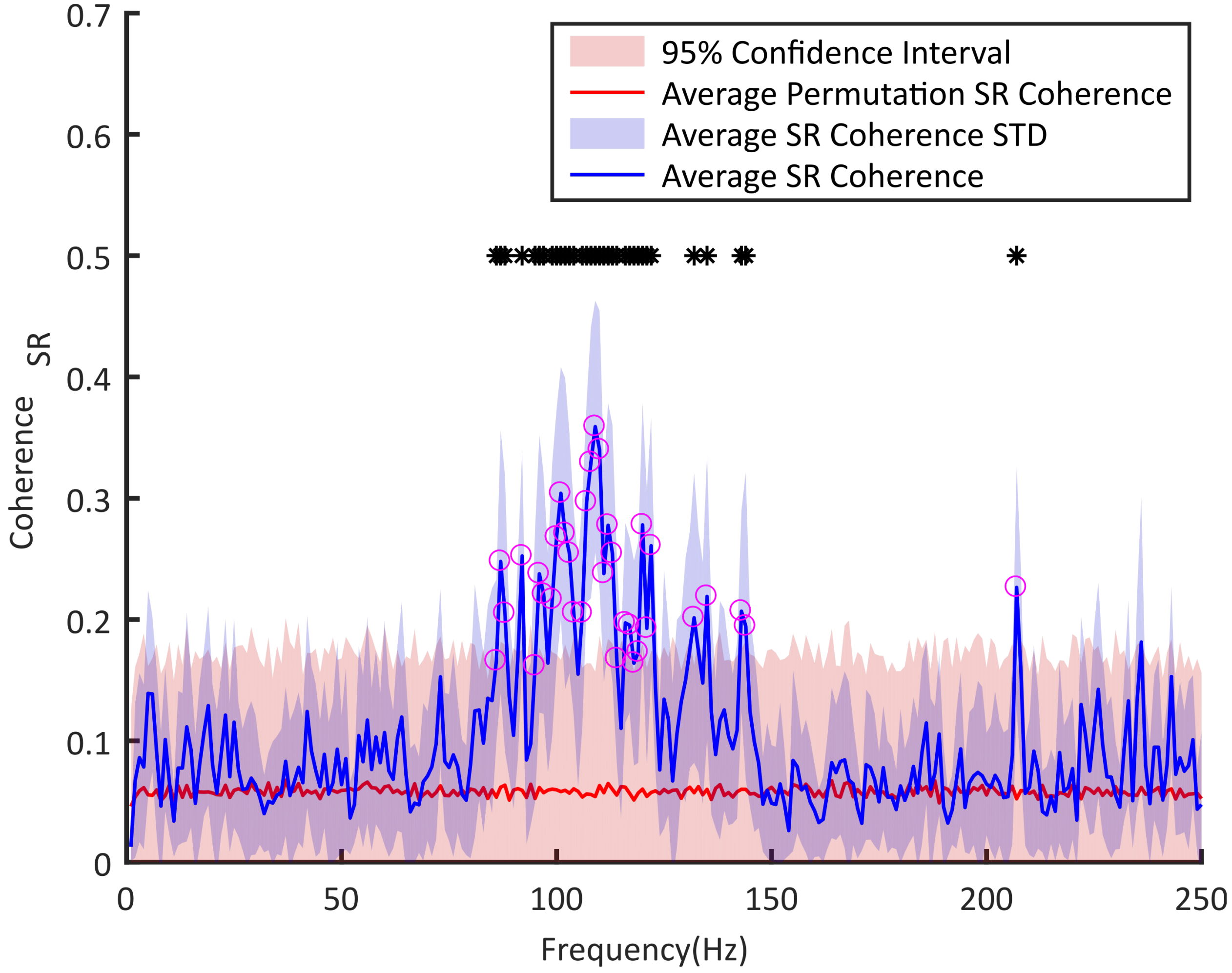

Supplementary Figure 2: Signal response (SR) coherence between the mechanical wing stimulus and the corresponding neural activity for a representative base localized unit. The mean SR coherence (blue line, standard deviation in shaded blue) of a representative base localized unit was greater than the 95% confidence interval (red shading, mean SR coherence of the randomly permuted spike times: red line) at various frequencies (Black stars and magenta circles represents the frequencies that had significant SR coherence values).

Supplementary Figure 3:

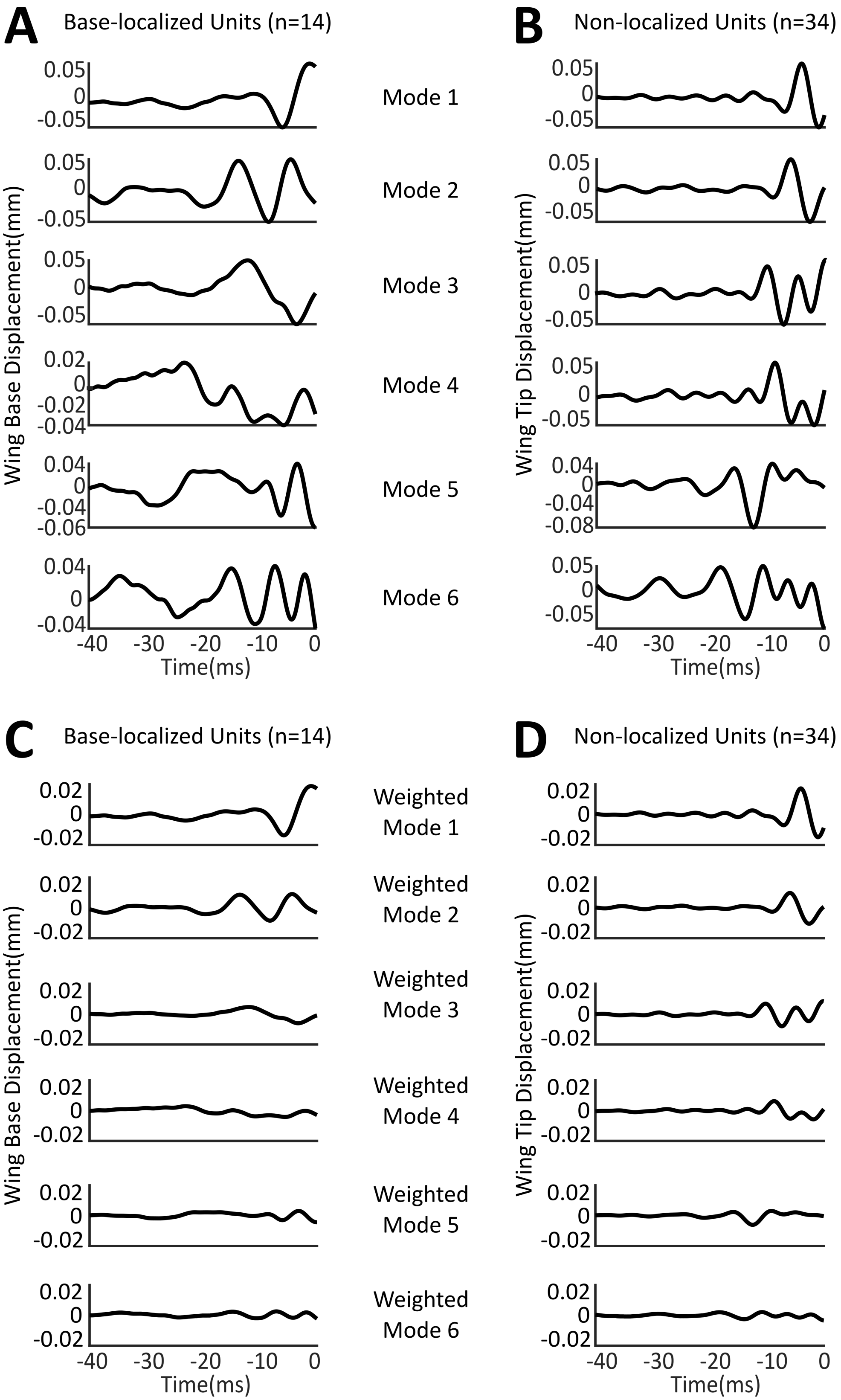

Supplementary Figure 3: SVD modes and weighted modes from the spike triggered averages (STAs) of the base-localized units (panels A & C) and non-localized units (panels B & D). The dominant six modes arising from the SVD analysis of the STAs of the base-localized units (n=14) and non-localized units (n=34) are shown in descending order (black traces in panels A & B). These modes were weighted by multiplying them by their corresponding normalized singular value (panels C & D). The singular values were normalized between 0 and 1 by dividing them by the sum of all the singular values. These modes were obtained by performing a singular value decomposition (SVD) on the STAs from each population of units. The six dominant modes were chosen because their singular values were greater than the maximum singular value resulting from the SVD on the priors of each population of units (see figure S4). Importantly, these modes reveal the major features shared across all STAs of each population. However, it appears that the first two modes contain the majority of the energy across the STAs.

Supplementary Figure 4:

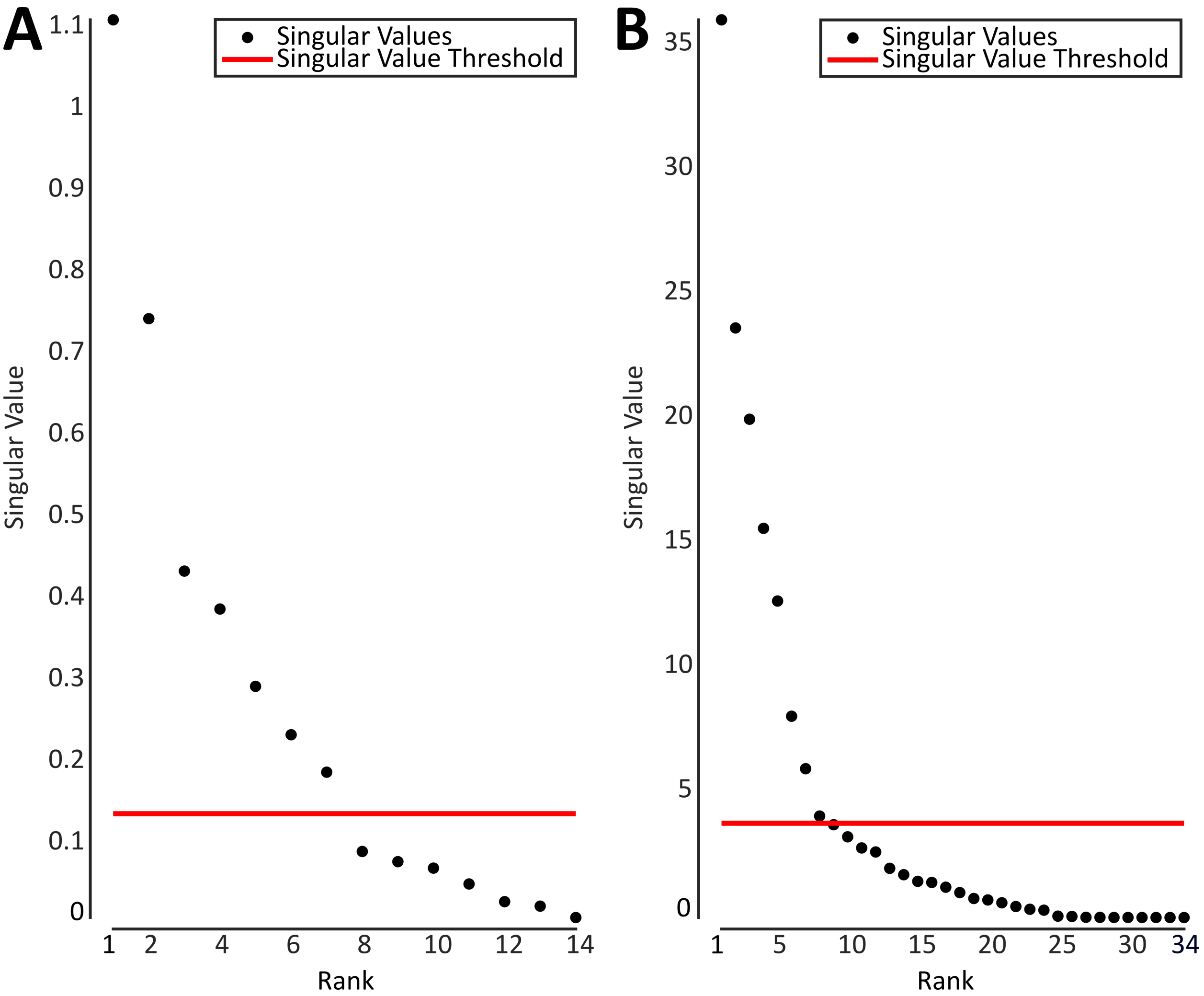

Supplementary Figure 4: Singular values from the SVD of the spike-triggered averages (STAs) of the base-localized units (panel A) and non-localized units (panel B). These singular values from the STAs of the base-localized units (n=14) and the non-localized units (n=34) are shown in descending rank (panels A & B). The horizontal red line is the threshold used to determine which singular values and their corresponding modes were significantly different than those obtained from a singular value decomposition (SVD) on the priors of the base-localized and non-localized units. This threshold is set at the maximum singular value arising from the SVD on the priors of each population of units. Eight singular values from the STAs of both populations of units exceed this threshold, however, we focused our analysis on the six dominant modes corresponding to the six largest singular values as they contained the majority of the energy across the modes.

Supplementary Figure 5:

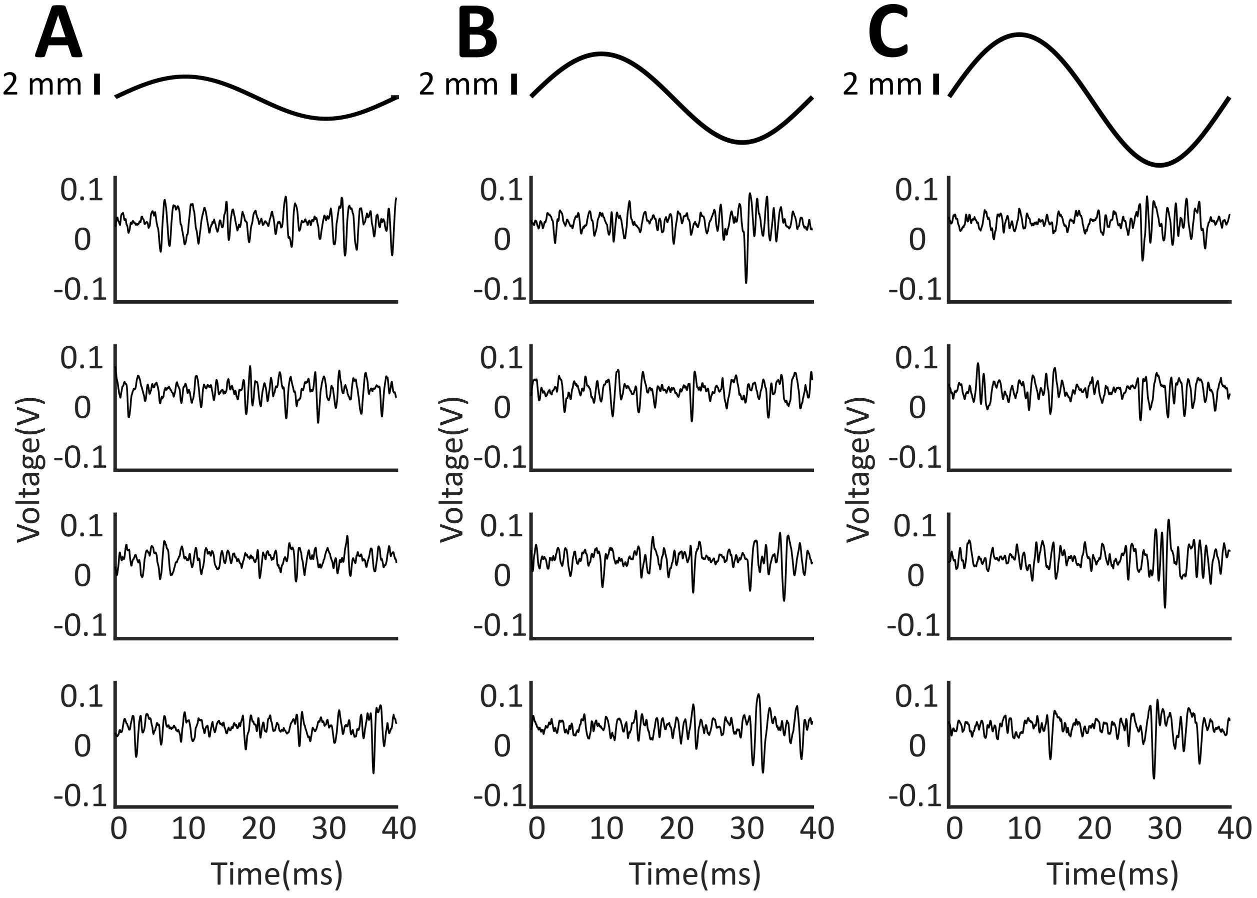

Supplementary Figure 5: Raw neural activity of the primary afferents of the wing nerve during varying amplitudes (panel A: 4.4 mm, panel B: 8.8 mm, and panel C: 13.2 mm) of sinusoidal displacements to the wing tip. Extracellular traces (lower four traces in each panel) during 40 ms cycles of sinusoidal displacements to the wing tip (upper trace in each panel) are shown for a representative wing nerve recording. Scale bar for the sinusoidal wing tip displacement is 2 mm.

Supplementary Figure 6:

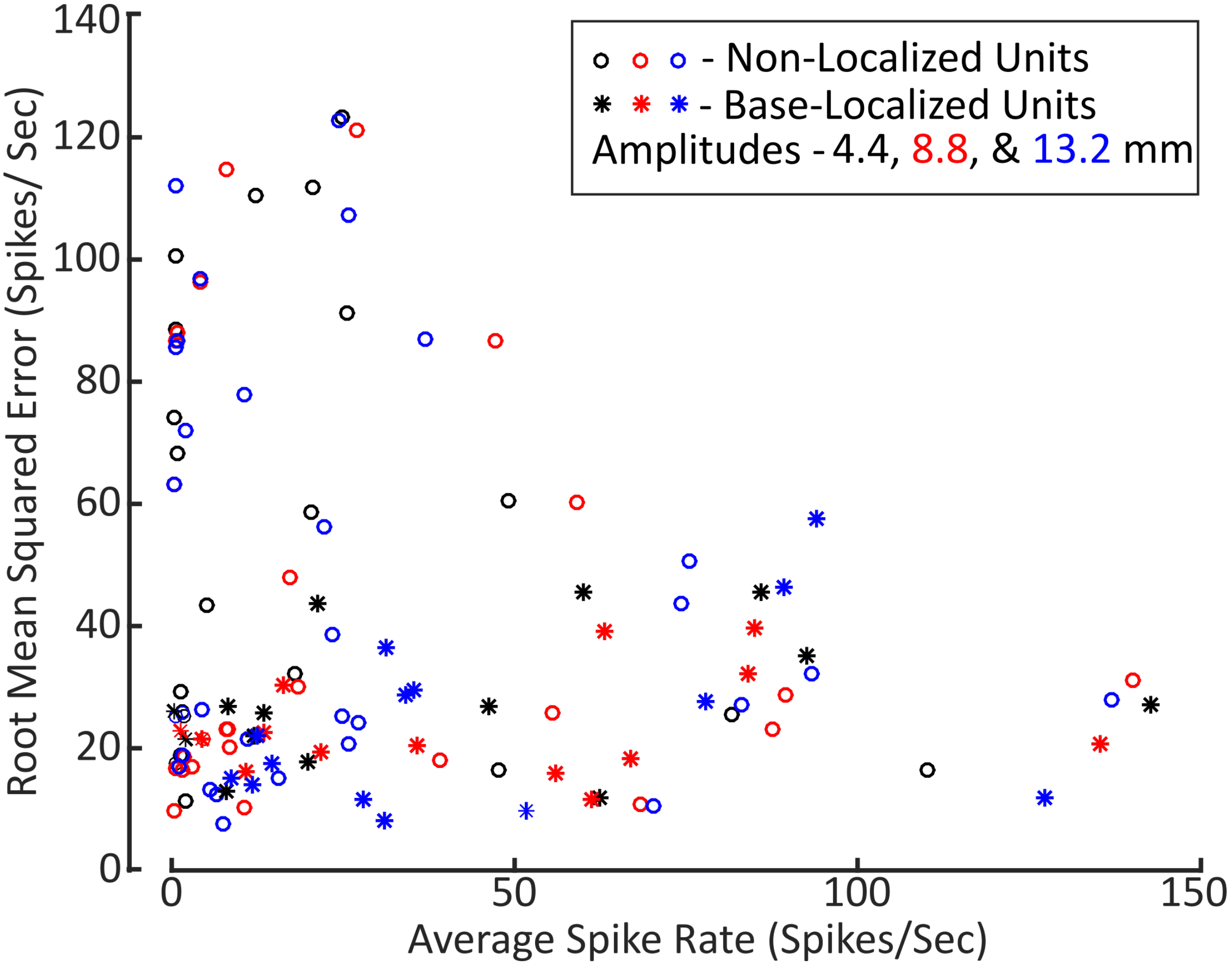

Supplementary Figure 6: Root mean square error (RMSE) between the recorded smoothened spike rate and the predicted spike rate during sinusoidal displacements of varying amplitude to the wing tip. Low RMSE values for all recorded units suggest that the one-dimensional spike prediction model could predict the spike rate of a unit. However, the ability to predict a unit's spike rate was generally better for units with high mean spike rate (Open circles; base localized units, stars; non-localized units, displacement amplitudes - 4.4 mm (black), 8.8 mm (red), and 13.2 mm (blue)).

Unit #	RMSE Sine Amplitude 4.4mm (Spikes/ Second)	RMSE Sine Amplitude 8.8mm (Spikes/ Second)	RMSE Sine Amplitude 13.2mm (Spikes/ Second)	Average Recorded Spike Rate During Sine Amplitude 4.4mm (Spikes/Sec)	Average Recorded Spike Rate During Sine Amplitude 8.8mm (Spikes/Sec)	Average Recorded Spike Rate During Sine Amplitude 13.2mm (Spikes/Sec)
1	45.51	39.05	28.61	60.01	63.01	34.00
2	43.63	15.95	57.45	21.25	56.01	94.01
3	26.88	18.28	27.73	46.26	67.01	77.76
4	22.08	30.40	29.45	12.00	16.25	35.25
5	45.57	39.64	46.36	86.01	85.01	89.26
6	21.58	22.74	14.07	2.00	1.25	11.75
7	35.18	32.21	36.55	92.51	84.01	31.25
8	25.98	21.55	17.44	0.25	4.50	14.50
9	25.69	19.45	8.15	13.50	21.75	31.00
10	17.86	20.33	11.73	19.75	35.75	28.00
11	26.92	22.60	22.17	8.25	13.50	12.50
12	12.89	16.16	15.04	8.00	10.75	8.75
13	27.17	20.69	11.77	142.77	135.27	127.27
14	11.90	11.73	9.78	62.51	61.26	51.76
15	91.25	60.36	50.74	25.50	59.01	75.51
16	58.67	25.72	43.79	20.25	55.51	74.26
17	112.83	112.76	112.04	0.00	0.00	0.50
18	68.39	47.84	38.63	0.75	17.25	23.50
19	111.69	86.62	107.16	20.50	47.26	25.75
20	87.09	86.74	86.61	0.00	0.50	0.75
21	43.43	30.06	25.13	5.00	18.50	24.75
22	110.39	114.74	87.03	12.25	8.00	37.00
23	18.14	18.11	17.04	0.00	0.00	1.00
24	18.85	16.94	20.82	1.25	3.00	25.75
25	74.02	74.35	72.01	0.25	0.00	2.00
26	24.94	24.95	24.98	0.00	0.00	0.00
27	85.74	85.83	85.58	0.00	0.00	0.50
28	17.59	16.56	13.34	0.50	1.50	5.50
29	25.72	25.72	25.27	0.00	0.00	0.50
30	9.79	9.65	9.73	0.00	0.25	0.00
31	60.46	28.60	27.23	49.01	89.51	83.01
32	78.09	78.01	56.26	0.00	0.00	22.25
33	25.96	20.24	25.85	1.50	8.50	1.50
34	32.24	17.97	24.11	18.00	39.01	27.25

35	25.16	21.56	15.06	1.75	4.75	15.50
36	11.43	10.35	7.67	2.00	10.50	7.50
37	63.61	63.29	63.24	0.00	0.25	0.25
38	17.28	16.74	12.41	0.00	0.50	6.50
39	NaN	NaN	NaN	0.00	0.00	0.00
40	16.55	31.19	27.79	110.26	140.02	137.02
41	100.58	96.39	96.91	0.50	4.25	4.25
42	31.27	22.98	21.57	0.00	8.25	11.00
43	88.48	88.07	77.95	0.50	0.75	10.50
44	123.31	121.17	122.75	24.75	27.00	24.25
45	25.43	23.06	32.24	81.76	87.51	93.26
46	20.43	18.65	18.90	0.00	1.75	1.50
47	16.40	10.92	10.50	47.76	68.26	70.26
48	29.23	23.11	26.37	1.25	8.00	4.50

Supplementary Table 1: Root mean square error between the average recorded spike rate and the predicted spike rate during sinusoidal displacements (amplitudes: 4.4 mm, 8.8 mm, and 13.2 mm) to the wing tip. Units above the solid dashed line are the base localized units, and below the solid dashed line are the non-localized units. Note that some units did not spike during the sinusoidal displacements of wing tip at all and hence we could not include them in this analysis.

Supplementary Movie:

Supplementary Movie 1: X-ray MicroCT reconstruction of the Wing Base highlighting some of the campaniform sensilla fields on the wing base and the tegula.

Supplementary Movie 2: High-speed stereo videography (upper left and right panel) and the resulting 3D reconstruction (lower panel) of the ventral surface of a representative moth wing during the onset of white noise mechanical stimulation to the wing tip. This movie consists of the first 500 frames captured by high-speed stereo videography at 1000 fps and correspondingly the 3D reconstruction of the ventral surface of the wing. The movie playback frame rate is 10 fps. While the mechanical stimulus is delivered in a single axis, this movie shows that more complex patterns of flexion still arise due to the structural complexities of the wing and elastic wave propagation.

Supplementary Materials and Methods:

1) X-ray microtomographic imaging

We dissected the fore- and hindwing pair along with some thoracic tissue and fixed the sample in 10% formalin ~24 hour at room temperature. After fixing, we dehydrated the samples through an alcohol concentration series of 15%, 30%, 50%, 60%, 75%, and 90% ethanol followed by 2 washes of 100% ethanol for 20 minute each. The wings were stained with 1% iodine in 100% ethanol solution for about 2 days at room temperature (tissue preparation adapted from [1]). After staining, they were critical point dried to better preserve the structure and mounted between two Styrofoam slices to hold them inside a plastic tube. The sample was imaged using SkyScan 1172 microCT scanner (Bruker MicroCT, Kontich, Belgium) and images were taken at 28kV source voltage, 157 µA current, 1800 millisecond exposure with a rotation step of 0.15 degree, and voxel size of 2.94 micron to get suitable images for analysis. The reconstructed data was loaded in Avizo 9.2 (FEI, Oregon, WA) for further analysis. We exported the analyzed data as animations and images.

High-speed videography and calibration

We captured the vertical displacement of the wing blade in response to the first 10-second segment of the white noise mechanical stimulus using two high-speed cameras (Miro-4M VR0308 and VR711, Vision research, Wayne, NJ) at 320 * 240 resolution, 1000fps, and 200-400 microsecond exposure. The volume of space that the wing would be held in was calibrated prior to experiments using a 20-point calibration object. 6 painted markers (3 wing base, 2 wing blade, 1 wing tip) on the ventral surface of the wing were digitized over 11,000 frames to reconstruct the wing motion using a custom written direct linear transformation software in Matlab (The Mathworks Inc., Natick, MA) [2]. All further analysis was done using custom written codes in Matlab. All digitized points were transformed from the global coordinate system of the calibration object to the wing specific coordinate system such that vertical displacements along the ventral direction were positive. Using this method, we reconstructed the vertical displacements of the wing blade for one repeat of the white noise mechanical stimulus.

We cross validated the vertical displacement of the wing tip from high speed videography with manual calibration of the motor lever tip displacement using a calibrated ocular micrometer. We measured the displacement of the motor lever tip at various input voltages (amplitudes ranging from 0 to 6.4 V) and determined the displacement output versus voltage input curve. We also cross-validated the calibration and the output measurements (in millimeters) of the high-speed cameras by recovering the expected length of a pre-measured rod.

2) Thermal mapping to identify the receptive field of each unit

We focally heated 5 specific locations on the wing using IR laser to map receptive fields of mechanosensory units. Test laser pulses of a lower intensity (10% duty factor) were used to adjust the position of the laser's focal point on the wing to one of five locations predetermined based on the campaniform sensilla locations seen in SEM images [3]. The five locations were: (1) the wing base, (2) the wing's leading edge, one third along the wing's length from the wing base, (3) the wing tip, (4) the interior wing blade, at the base of medial vein 3 and, (5) a control location: in which the laser was turned on but not localized

on the wing surface to ensure that the laser itself was not influencing the neural activity of the primary afferents.

We used changes in firing rate to determine whether the recorded unit responded to the laser stimuli. Each laser location was heated 5 times, resulting in 25 firing rate responses across the 5 locations. The average firing rate was projected onto each of these 25 responses. A coherent and large response throughout the 5 applications of heating to a location indicates that the unit projects from that location on the wing (figure S1).

3) Selection of wing base neuronal units through signal response coherence analysis

A Signal response (SR) coherence reflects the amount of mutual information between the stimulus and the neuronal activity of a unit [4]. We used this measure to select units that encode information about the stimulus for further analyses (figure S2).

For each unit, we convolved the spike time with a 1ms Gaussian window and calculated the magnitude squared coherence between the spike train and the stimulus (vertical displacement) over the frequency range of 1 to 250 Hz using a window length of 1 second. Next, we used bootstrapping method to determine the SR coherence between randomized spike times and the mechanical stimulus. We randomly permuted spike times for each unit, a total of 1200 times (40 permutations for each of the 30 repeats of the white noise stimulus) and calculated the coherence measure for each permutation. This allowed us to build a distribution for the SR coherence of randomly permuted spike trains, with a mean and a 95% confidence interval. We then compared the mean SR coherence for the recorded spike trains (n = 10) and included only those units whose mean SR coherence was higher than the 95% confidence interval of the randomly permuted spike trains at any frequency.

4) SVD modes of the STAs of the base-localized and non-localized units

A singular value decomposition (SVD) was performed in Matlab on the spike-triggered averages (STAs) of the base-localized (n=14) and non-localized units (n=34) to extract the dominant shared modes across the STAs. The STAs of each population of units

were stored in a matrix in which the columns represented the STA number and the rows were the displacement values of the STA at given sample points (sampling rate of 40 kHz). The duration of the STA was 40 ms, and therefore, each row of these two matrices was 1600 indices. From the SVD of these matrices, the columns of the resulting 'U' matrix were the modes and the diagonal values of the 'S' matrix were the singular values. This process was repeated for the priors of the base-localized and non-localized units. The six dominant modes from the STAs of both the base-localized and non-localized units were selected and focused upon because their corresponding singular values were greater than the largest singular value obtained from a SVD on the priors of both sets of units. These six dominant modes of each population of units were weighted relative to one another by multiplying them by their corresponding normalized singular value. The singular values were normalized between 0 and 1 by dividing them by the sum of all the singular values. The weighted modes reveal that the first two dominant modes contain the majority of the energy across the STAs.

References:

1. Metscher BD. 2009 MicroCT for comparative morphology : simple staining methods allow high-contrast 3D imaging of diverse non-mineralized animal tissues. *BMC Physiol.* **9**. (doi:10.1186/1472-6793-9-11)
2. Hedrick TL. 2008 Software techniques for two- and three-dimensional kinematic measurements of biological and biomimetic systems. *Bioinspir. Biomim.* **3**. (doi:10.1088/1748-3182/3/3/034001)
3. Dickerson BH, Aldworth ZN, Daniel TL. 2014 Control of moth flight posture is mediated by wing mechanosensory feedback. *J. Exp. Biol.* **217**, 2301–2308. (doi:10.1242/jeb.103770)
4. Borst, A. Theunissen FE. 1999 Information Theory and neural coding. *Nat. Neurosci.* **2**, 947–957.
